# Parental Divorce in Childhood and the Accelerated Epigenetic Aging for Earlier and Later Cohorts: Role of Mediators of Chronic Depressive Symptoms, Education, Smoking, Obesity, and Own Marital Disruption

**DOI:** 10.1007/s12062-023-09434-5

**Published:** 2023-10-31

**Authors:** Jung Ki Kim, Thalida Em Arpawong, Eric T. Klopack, Eileen M. Crimmins

**Affiliations:** 1 Leonard Davis School of Gerontology, University of Southern California, Los Angeles, CA 90089-0191, USA

**Keywords:** Cohort Difference, Childhood Adversities, Epigenetic Aging, DunedinPACE

## Abstract

We examine effects of parental divorce on epigenetic aging in later adulthood for two birth cohorts: one born in the early 20th century and the other born in the later 20th century. Using data from the Health and Retirement Study (n = 1,545), we examine the relationship between parental divorce in childhood and accelerated epigenetic aging in older adulthood as indicated by the Dunedin methylation Pace of Aging score. We assess how this relationship is mediated by chronic depressive symptoms, education, lifetime smoking, body mass index (BMI), and an older adult’s own divorce. The mean age of the earlier cohort is 85.8 (SD = 3.9) and that of the later cohort is 60.2 (SD = 2.8). We find that parental divorce was related to faster aging in the later-born cohort, and that 56% of this relationship (b = 0.060) was mediated by chronic depressive symptoms (b = 0.013), lower education levels (b = 0.005), and smoking (b = 0.019). For the earlier cohort, there was no effect of parental divorce on epigenetic aging. Parental divorce in childhood may have lasting effects on later-life health, as reflected in the rate of epigenetic aging. However, the effects and mechanisms of this relationship differ across cohorts living in different social environments.

## Introduction

Children of divorced parents are more likely to experience negative health outcomes such as depression, weakened immune response, and premature mortality (Auersperg et al., 2019; Nunes-Costa et al., 2009; Tucker et al., 1997). The lifecourse perspective is a useful framework for understanding how early traumatic experiences such as parental divorce can affect later-life health outcomes. This perspective highlights that events during formative years can exert lasting effects on well-being and health across the entire lifespan, shaping physiological systems, social behaviors, and behavioral patterns over time.

Recent studies have suggested the potential link between parental divorce and biological aging through epigenetic changes. Epigenetic changes are altered gene expression through DNA modifications, potentially linking early life stressors, such as parental divorce, to accelerated aging and increased risk for age-related diseases in later life (Belsky et al., 2022; Joshi et al., 2023). Epigenetic aging, which reflects the cumulative impact of lifelong biological and environmental stressors, may provide health implications for our understanding of the impact of parental divorce on children’s health in later life. It may capture unrecognized biological processes occurring earlier in life relevant to health outcomes such as frailty, morbidity, and mortality (Crimmins et al., 2021). At the same time, the life-course perspective emphasizes the importance of social, environmental, and historical contexts in shaping individual trajectories over time. This means the links between parental divorce and later-life epigenetic aging may vary by birth cohorts whose lives unfold through different social environments.

Considering historical shifts, it is plausible that the association between parental divorce and later epigenetic aging might be more pronounced for those who grew up in the earlier years of the past century than for those raised in later years. During the first half of the century, divorce was less common and carried greater social stigma (Greenwood & Guner, 2009; Plateris, 1973; Wilcox, 2009). However, in the second half of the century, as societal attitudes shifted and divorce became more accepted and feasible due to women’s increased labor force participation (Waite, 1981) and the adoption of no-fault divorce laws (Jacob, 1988; Oren, 2018), its potential impact on children’s health might have lessened.

The effects of parental divorce may lead to changes in social and environmental contexts that, in turn, affect health behaviors and physiological stress responses throughout an individual’s development. In particular, parental divorce may lead to chronic mental health issues for children (Amato, 2001a; Auersperg et al., 2019; Cherlin et al., 1998; O’Connor et al., 1999; Rodgers, 1994; Sands et al., 2017; Singer et al., 2022), strained relationships with parents, reduced time spent with parents, adoption of unhealthy behaviors (Amato & Keith, 1991a, b; Felitti et al., 1998; Thomas & Högnäs, 2015), lower educational attainment (Amato & Keith, 1991a, b; Brand et al., 2019), financial instability at formative points in life, and increased likelihood of divorce in children’s own marriages (Huurre et al., 2006).

Like the general effect of divorce on children, the effect of each of these mediators may differ by cohort because of societal changes over time. Childhood experience of parental divorce could lead to childhood depression (Aseltine, 1996), persisting into adulthood (Ross & Mirowsky, 1999; Sands et al., 2017). Rising divorce rates could weaken the link between parental divorce and adult depression. Children with divorced parents may struggle with education due to reduced motivation and parental support, additional familial responsibilities, and a need to work to contribute financially (Amato, 1991b; Demir-Dagdas et al., 2018), which may be particularly pronounced in more recent cohorts who have faced increased expectations for higher education. Coping mechanisms like smoking and overeating (Thomas & Högnäs, 2015; Tucker et al., 1997; Wolfinger, 1998) may vary by cohort because of changing societal norms. For instance, post-war era children of divorced parents may have turned to smoking, which was less common and acceptable than it had been in earlier years, to cope with stress and maladaptation, while pre-war children may have responded by overeating and becoming obese when obesity was not common (Cutler et al., 2003). Children of divorcees are more likely to experience divorce in their own marriage (Huurre et al., 2006; Tucker et al., 1997), with such divorce having its own adverse effects on health in later life. The connection between parental divorce and one’s own divorce may vary by cohort and be less strong when divorce is more common (Easterlin et al., 1993). Additional and alternative mechanisms are considered in the Discussion in light of the findings.

This study examines the link between parental marital disruption in childhood and accelerated epigenetic aging in later life, and how this link may differ by cohort. It also considers the mediators of chronic depressive symptoms, education, lifelong smoking and obesity, and own divorce. We seek to answer two questions: (1) Does the relationship between parental divorce and epigenetic aging differ by birth cohort, and (2) how do the mechanisms linking parental divorce to accelerated epigenetic aging differ by cohort?

We hypothesize that the effect of parental divorce on accelerated epigenetic aging will be greater on cohorts born earlier. We also hypothesize that lower educational attainment and lifelong smoking would be more likely to mediate the relationship between parental divorce and epigenetic aging in later cohorts, while chronic depressive symptoms, obesity, and own divorce will be mediators for earlier cohorts.

## Data and Methods

### Data

We use data from the Health and Retirement Study (HRS), which is a nationally representative longitudinal survey of the U.S. population 51 years and older and their spouses. It began in 1992 for those born between 1951 and 1961 and those born before 1924 were added in 1993. It continues with biennial waves and new cohorts entering every 6 years (Sonnega et al., 2014). The HRS is a household survey based on complex sampling frames to enhance the national representativeness of the sample. It oversamples African American and Hispanic households to provide sufficient statistical power for subpopulation analysis (Sonnega et al., 2014). We derive an epigenetic aging measure from the HRS venous blood subsample (N = 4,108) collected in 2016. We derived parental divorce from the Life History Mail Survey collected in 2015 and 2017 and other variables as collected from 1992 to 2016.

We consider two birth cohorts: those born between 1916 and 1935, and those born between 1951 and 1965. The earlier cohort grew up in a period of low divorce rates. The later cohort grew up in a period of increasing divorce rates as well as economic prosperity and societal change. We assess the effect of parental divorce occurring before a child reached age 16; thus, the timing of parental divorce for the earlier cohort spans from 1916 to 1950 and that of the later cohort from 1951 to 1980.

Of the 4,018 respondents with epigenetic aging measures, 2,209 were in one of our two birth cohorts. Of these 2,209, we excluded 629 from our analysis because they did not participate in the Life History Mail Survey or had missing information on parental divorce. We excluded an additional 35 individuals due to missing data on other variables. This yielded an analysis sample of 1,545 persons, 470 of whom were born from 1916 to 1935, and 1,075 who were born from 1951 to 1965 (Table 1). The mean age of the earlier cohort was about 86 in 2016, the year of the venous blood sample we used to assess epigenetic aging, while that of the later cohort was about 60. The 664 HRS respondents in these cohorts whom we excluded from our analysis because they had some missing information had, on average, higher rates of epigenetic aging as indicated by DunedinPACE than the analytic sample of 1,545 persons (mean 0.81 vs. 0.75, p = .002; minimum 0.35 vs. 0.15). We conducted Little’s MCAR test to determine whether missing values are completely at random (MCAR). Using the Stata user-written package, mcartest, and a series of *x*^2^ tests with the missingness of the variables with gender, we see that all variables other than parental divorce are missing completely at random, and the missingness of parental divorce is not completely random (p = .003). Furthermore, the absence of data on parental divorce is significantly associated with all mechanisms (p < .05) except for BMI among the earlier cohort, and it shows significant associations with chronic depressive symptoms and education (p < .001) among the later cohort. As we examined the effect of parental divorce on epigenetic aging, through these mediators, it is worth noting that the missing information about parental divorce and its relationship with other mechanisms could potentially influence the effects differently for the two cohorts. Thus, sample selection based on the availability of parental divorce which was from HRS subsamples of the Life History Mail Survey may have affected the study findings. The dropped respondents were also, on average, younger (66.2 vs. 67.5, p = .018) and less likely to be female (54.1%% vs. 60.5%, p = .005).

## Measures

### Accelerated Epigenetic Aging

Previous studies of the relationship between childhood adversity and DNA methylation have used first- and second-generation epigenetic clocks such as Horvath, Hannum, PhenoAge and GrimAge (Hamlat et al., 2021; Hughes et al., 2018; Joshi et al., 2023; Tang et al., 2020; Zannas et al., 2015). We use a third-generation DNAm measure, Dunedin methylation pace of aging (Belsky et al., 2022) as used in recent studies (e.g., McCrory et al., 2022, Schmitz et al., 2023). This is an epigenetic measure of the rate of change (or pace) of an individual’s biological aging using elastic net regression of 19 clinical and biological indicators observed at four time points from age 26 to 45 (Belsky et al., 2020; Elliott et al., 2021). DunedinPACE is estimated in years of aging per chronological year, indicating accelerated/decelerated aging (Faul et al., 2023). This measure has enhanced test-retest reliability relative to earlier measures by only including the most reliable probes (Sugden et al., 2020). DunedinPACE in HRS was calculated by using the code provided at https://github.com/danbelsky/DunedinPACE. DunedinPACE is useful for gauging the relationship of parental divorce and speed of epigenetic aging at older ages, as well as how this relationship is affected by mediating behaviors; this is because it is strongly associated with health outcomes and consequences of hardships experienced throughout life (Belsky et al., 2022; Faul et al., 2023; Graf et al., 2022).

### Parental Divorce

We coded respondents who reported that their parents divorced before they reached the age of 16 as having experienced parental divorce.

### Mediators

#### Chronic Depressive Symptoms.

We used the CESD-8 scale to measure depressive symptoms. The Center for Epidemiologic Studies Depression Scale (CESD) is a well-validated and commonly used measure to assess depressive symptoms in various age groups, including older adults (Radloff, 1977). HRS uses the CESD-8, a shorter version of the original CESD which has been shown to be reliable in the older population, with Cronbach’s alpha coefficients of 0.81 in the HRS. The eight items of the CESD-8 scale ask respondents how much during the past week they (1) felt depressed; (2) felt that everything they did was an effort; (3) had restless sleep; (4) were happy; (5) felt lonely; (6) enjoyed life; (7) felt sad; or (8) could not get going. We reverse-coded items (4) and (6) to create an additive scale of depressive symptoms. We calculated this scale for respondents from 1994 to 2016 and averaged the available scores (because the 1992 HRS did not have comparable questions on depressive symptoms, we excluded it from our measure).

#### Education.

We assessed educational attainment in years of school completed, ranging from 0 to 17 years, where the upper limit of 17 years denoted the attainment of a post-graduate degree.

#### Smoking.

We measured smoking pack years, the reported number of cigarette packs by lifetime years of smoking (NCI, 2023). It is calculated by multiplying the reported average daily number of cigarette packs smoked and the duration of smoking in years (Haghani et al., 2020; Klopack et al., 2022).

#### Own Divorce Experience.

We coded respondents who had a divorce in their own lifetime as 1 for ‘yes’ or 0 for ‘no.’

#### Highest BMI.

We used the highest BMI value calculated by self-reported height and weight to indicate obesity. We used the highest rather than mean BMI over all waves because lifetime maximum BMI has been found to be a more valid predictor of health outcomes and is more comparable across cohorts (e.g., Singer et al., 2018, Taghizadeh et al., 2015). Our sensitivity analysis showed that the median difference between maximum and mean BMI is only about 1.7, and using mean or current BMI did not change our results.

### Control Variables

We included respondent age, gender and race/ethnicity (non-Hispanic White, non-Hispanic Black, and Hispanic) in our model to control for their potential effect on parental divorce (Bumpass, 1984; Hetherington & Stanley-Hagan, 1999; Lee & McLanahan, 2015; Leopold, 2018).

### Analysis

We began with descriptive statistics for the two birth cohorts. Next, we conducted ordinary least squares (OLS) regression analysis to examine the effect of parental divorce when only controlling for demographic characteristics. We then included all proposed mediators to examine whether parental divorce influences epigenetic aging and how it changes with other potential mediators. We then conducted a formal mediation analysis, using a method for comparing indirect effects based on the product of coefficients. To compute indirect effects from multiple models including both continuous and binary mediators, we standardized coefficients. We used the command binary mediation in Stata version 16 to obtain coefficients to compute the total, direct, and indirect effects between parental divorce and DunedinPACE with our mediator variables and to estimate how much mediators explain the relationship. For the mediation analysis, we multiplied the DunedinPACE score by 100 to make the coefficients larger for readability. We did mediation analysis separately for the two cohorts. All models included age, gender, and race/ethnicity.

## Results

Table 1 shows that 9.81% of the earlier cohort and 15.70% of the later cohort in our sample experienced parental divorce by the time they were 16 years old (p < .01). The later cohort reported greater depressive symptoms (CESD-8 score of 1.41 vs. 0.98, p < .001), more years of education (13.85 vs. 12.72, p < .001), lower cigarette pack years (9.29 vs. 13.50, p < .001), higher BMI (31.36 vs. 29.35, p < .001), and higher rates of their own divorce (20.98% vs. 13.73%, p < .01).

Table 2 shows the OLS regression results for earlier and later cohorts separately. For the earlier cohort, parental divorce was not related to faster epigenetic aging. The fit statistics showed that 9% of the variance in DunedinPACE was explained by the model’s predictors (adjusted R^2^ = 0.05 for M1, 0.09 for M2) and RMSE and MAE showed that the models’ predictions were over 10% of the range of DunedinPACE (RMSE = 15.28 for M1, 14.93 for M2; MAE = 11.92 for M1, 11.43 for M2). For the later cohort, parental divorce was significantly related to faster aging in the model controlling only for age, gender and race/ethnicity (b = 2.876, 95% CI: 0.441, 5.311) but not in the model including all proposed mediators. Lower education (b=−0.934, 95% CI: −1.264, −0.604), more smoking (b = 0.158, 95% CI: 0.110, 0.207) and higher BMI (b = 0.691, 95% CI: 0.584, 0.799) were related to accelerated epigenetic aging among members of the later cohort. This suggests an indirect association between parental divorce and epigenetic aging, with education, smoking, and BMI mediating the relationship. The fit statistics showed improved performance for the later cohort, better explaining the variance in DunedinPACE (adjusted R^2^ = 0.13 for M3, 0.31 for M4) and producing predictions (RMSE = 14.56 for M3, 12.93 for M4; MAE = 11.26 for M3, 10.12 for M4).

**Fig. 1:**
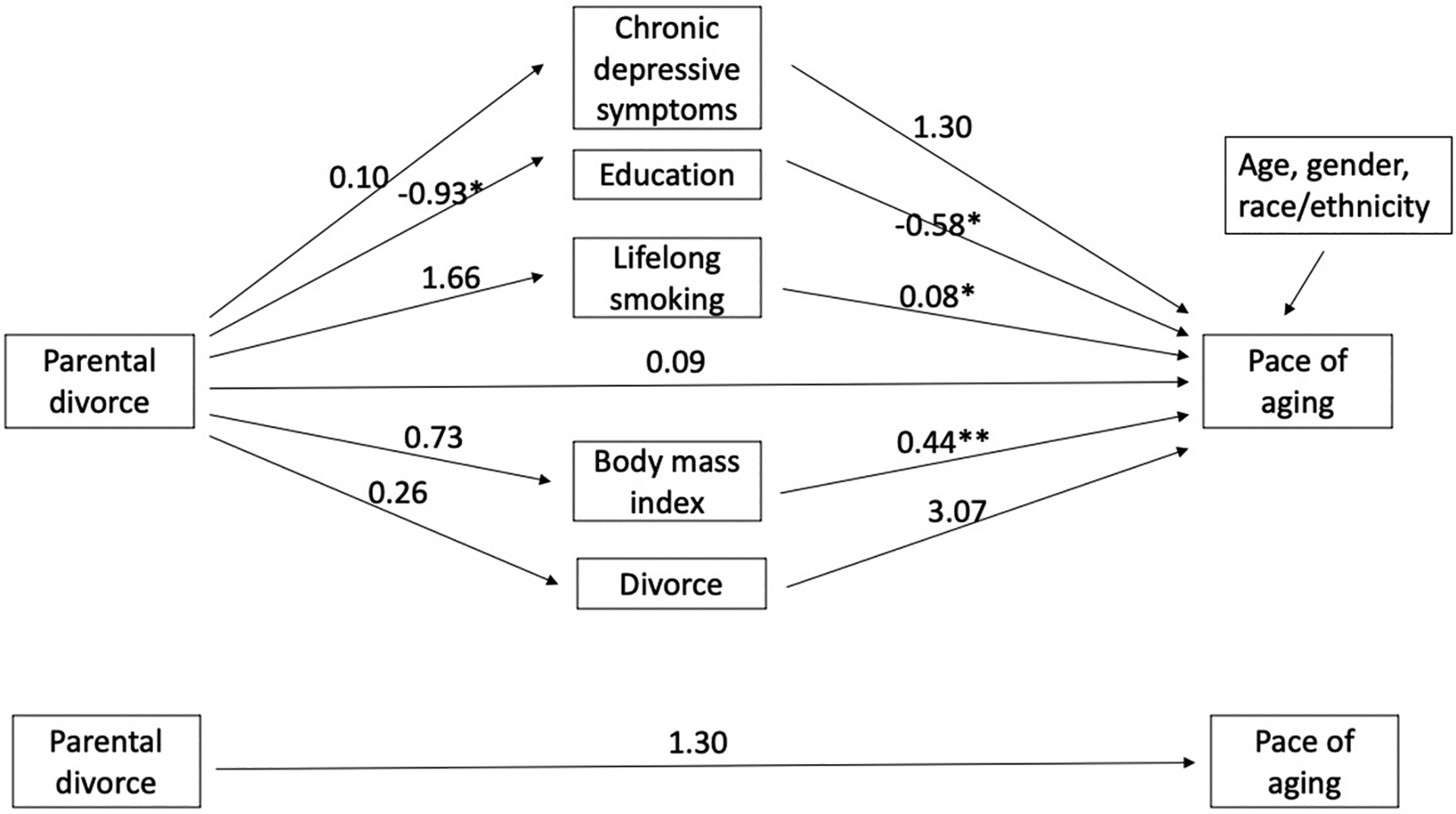
Unstandardized coefficients from mediation models between parental divorce and epigenetic aging: earlier cohort

**Fig. 2:**
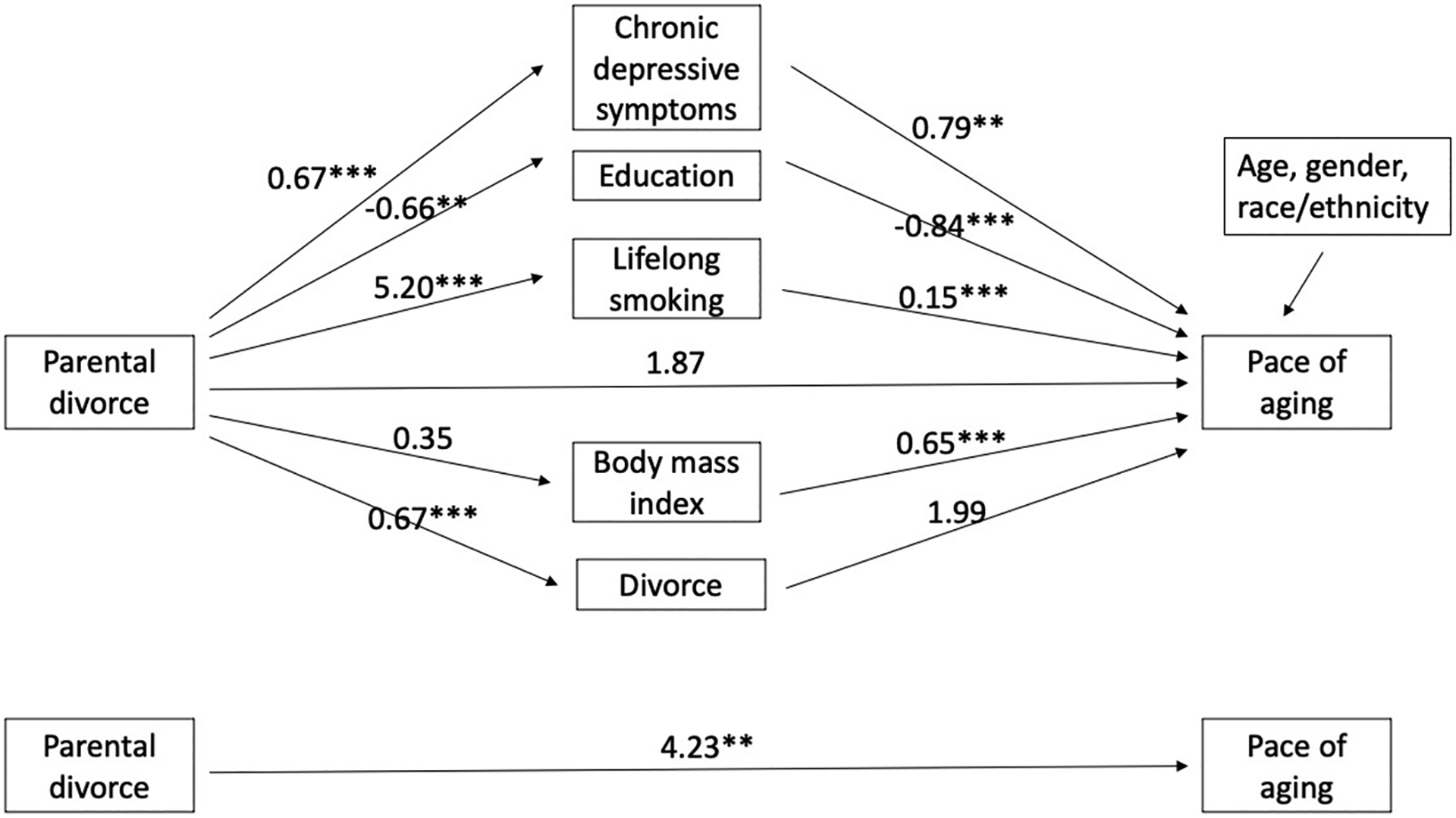
Unstandardized coefficients from mediation models between parental divorce and pace of aging: later cohort

We conducted formal mediation analysis on the role of mediators in the association of parental divorce with epigenetic aging. Figures 1 and 2 show the coefficients from regression equations and control for all mediators for earlier and later cohorts separately. Among earlier cohort members (Fig. 1), parental divorce was not related to epigenetic aging either in the models with (above) or without mediators (below), nor were any of the first paths significant (parental divorce to mediators) except parental divorce to education. Among the later cohort members (Fig. 2), parental divorce was significantly related to epigenetic aging in the model without mediators (b = 4.23, p < .01) but was not directly related to epigenetic aging in the model with all mediators. Parental divorce was significantly related to all mediators except BMI, including greater chronic depressive symptoms (b = 0.67, p < .001), lower education (b=−0.66, p < .01), more lifelong smoking (b = 5.20, p < .001) and own divorce (b = 0.67, p < .001). In the second path (mediators to DunedinPACE), greater depressive symptoms, lower education, more smoking, and higher BMI were linked to accelerated epigenetic aging.

We estimated the total, direct and indirect effects of parental divorce on epigenetic aging via multiple mediators (Table 3). For the earlier-born cohort, there was no relationship between parental divorce and epigenetic aging. For the later cohort, the indirect effect was estimated to be 0.060 (bootstrapped SE: 0.016, 95% CI: 0.029, 0.090). This was mainly a result of the effects of parental divorce on aging through chronic depressive symptoms (b = 0.013, 95% CI: 0.005, 0.023), education (b = 0.005, 95% CI: 0.004, 0.025), and smoking (b = 0.019, 95% CI: 0.008, 0.031). Most (56%) of the effect of parental divorce on epigenetic aging was through its indirect effect. In other words, rather than being a direct effect on aging, the effect of parental divorce on epigenetic aging was significantly mediated by other variables.

## Discussion

This study examined the links between parental divorce in childhood and epigenetic aging in later life for two birth cohorts who lived the formative parts of their lives during different times. Our study demonstrates how the effect of early-life experience on later-life health can differ by cohort, both directly and through a range of social and environmental mechanisms that may change over time. By applying the lifecourse paradigm to our analysis, we were able to observe the differential effect for early and later cohorts and to identify several potential mediators of the relationship between parental divorce and epigenetic aging. These mediators, including chronic depressive symptoms, education, lifetime smoking, and BMI, were selected based on theoretical and empirical rationale while considering possible influence of cohort differentials. For example, drinking was not included in our models after the initial testing. These selected mediators are significant in representing key psychological, socioeconomic, and behavioral dimensions that reflect both individual and contextual influences that operate across the lifecourse. They highlight the need for a comprehensive approach to understanding the long-term effects of early-life stressors on later-life health. While previous studies on childhood adversity and health have suggested that parental divorce may increase the rate of aging in later life (Joshi et al., 2023), we examined how this effect differed by cohorts and mediating relationships. We found that parental divorce was significantly associated with faster epigenetic aging among those who were raised after 1950 but not for those raised in an earlier period.

Our initial hypothesis was that the effect of parental divorce on later-life epigenetic aging would be stronger for the earlier cohort. We assumed that the growing prevalence of divorce might have attenuated its negative impact over time. Our findings suggest that the consequences of parental divorce may have a stronger effect on later-life health for the later cohort. It is possible that the level of stress among children of divorce may be greater among the later cohort due to the changing nature and process of parental divorce. Divorces in earlier years may have occurred due to more unstable, adversarial and unhealthy marriages, with one partner likely found to be “at fault” for having committed adultery, abandonment, or cruelty. Divorce in the 1920s to 1940s might have been more common in marriages involving significant discord between parents or physical or emotional abuse. In such marriages, children may have benefitted from parental divorce (Amato, 2001b). Many of the children in 1950s to 1980s, on the other hand, might have been in less-toxic family situations, and less likely to benefit from but more likely to be harmed by parental divorce. Passage of no-fault divorce laws starting in 1970 made divorce easier and more frequent and may have led to dissolution of marriages which earlier had been considered to be “good enough.” Children in relatively stable families in the later cohort might have been more acutely affected by divorce and subsequently suffered greater depressive symptoms.

This study is based on the nationally representative population, thus it is expected to apply to other people from the same cohorts as our study. While the results contribute to our understanding of cohort differences in the relationship between parental divorce and children’s later-life epigenetic aging, it is advisable to approach generalization of these findings to other cohorts and countries with information on context. The variation in the relationship across cohorts may be contingent upon specific cohort characteristics and diverse demographic and social circumstances encountered including divorce norms, practice, rates and expectation about divorce.

By framing our analysis within the lifecourse paradigm, we are able to highlight the complex relationships between parental divorce, social and environmental contexts, and epigenetic aging. We found that chronic depressive symptoms, lower education, and smoking were important mediators of the relationship between parental divorce and epigenetic aging in the later cohort. These factors may reflect broader patterns of disadvantage and adversity that accumulate over time and may be particularly salient in shaping epigenetic aging of individuals who experience early-life stressors.

Our findings show that lower education mediated the link between parental divorce and faster epigenetic aging among the later cohort, supporting previous research. This suggests that parental divorce may have lowered a child’s education by reducing interest in school, generating more stress that made school more difficult, or even led children to quit school because of the family’s financial situation (Amato, 2001a, 2010; Frisco et al., 2007; Härkönen et al., 2017). This result may be related to changes in the labor market and educational opportunities. In later years, more people obtained higher education and job growth was greater for those with advanced education, leading to a disadvantage for those with lower educational levels, including children suffering from the effects of a divorce. Lower educational attainment for children of divorced parents might have negatively impacted their health in many ways over later life (Dalgard et al., 2007; Ross & Wu, 1995; Zajacova & Lawrence, 2018).

The mediating effect of smoking on the relationship between parental divorce and epigenetic aging among the later-born cohort might be related to societal changes in attitudes towards smoking. Smoking was much more prevalent in earlier decades and may have been viewed as a more socially acceptable way of coping with stress. In the second half of the 20th century, there was growing awareness of the negative health consequences of smoking (Apollonio et al., 2016), and societal attitudes towards smoking began to shift. Smoking for children of divorced parents in the later cohort may be an indication of more severe stress and maladaptation. Parental divorce may have a negative lingering effect into adulthood through the maintenance of smoking patterns adopted in early life (Lindström & Rosvall, 2020; Tucker et al., 1997; Vander Weg, 2011). Such smoking patterns could lead to faster epigenetic aging in later life. Persons in the later cohort who experienced parental divorce might have been at higher risk of chronic depression and resource constraints, leading to increased stress and the adoption of smoking. This suggests that mediators do not operate separately in increasing epigenetic aging.

We used DunedinPACE to measure epigenetic aging in our study, but, to verify our results, we tested the effects as measured by other epigenetic clocks. We found similar associations with PCGrimAge and PCPhenoAge as we did with DunedinPACE, but found no relationship between parental divorce and epigenetic aging when using PCHorvath1 and PCHannum.

The study’s findings, while specific to two birth cohorts, hold broader implications for research, public health interventions, and clinical settings. First, the study findings highlight the need to account for cohort-specific experiences and historical contexts when examining the relationship between early life experience and later-life epigenetic aging. These findings also underscore the importance of incorporating lifecourse perspectives to unravel the intricate interplay of social, environmental, and individual factors on health over time. Also, the identification of mediators like chronic depressive symptoms, education, and smoking emphasizes the potential for targeted interventions tailored to specific cohorts. Such interventions, ranging from mental health support and educational initiatives to smoking cessation programs, could help mitigate the adverse health effects of parental divorce. Clinically, the study findings prompt healthcare providers to adopt a context-based approach when addressing the health consequences of early-life stressors. Recognizing that the impact of parental divorce may vary across cohorts, clinicians can tailor discussions and strategies to patients’ specific cohort characteristics and life circumstances, ultimately enhancing their approach to promoting healthier aging for diverse populations.

Our study has some limitations. We did not control for parents’ marital status and living arrangements after divorce, the level of conflict that preceded divorce, or parental psychological and behavioral status. We lacked information on these, but all could influence the level of impact of parental divorce on later health of children (Amato, 2001a). Also, we did not have information on the exact age at which people experienced parental divorce, which could also influence the role of the mediating factors we examined (Thomas & Högnäs, 2015). We examined cohort differences in the relationship between parental divorce and children’s later health, considering that these cohorts also varied significantly by age, potentially affecting their epigenetic aging experiences differently. Additionally, we acknowledge the potential impact of differential mortality between cohorts on our study’s findings. Due to the inherent age disparities among the cohorts, a larger number of respondents from our first cohort had died before our analysis year in 2016. The implications of this differential mortality are multifaceted; those who were no longer alive could have encountered various adverse health behaviors, greater health issues, and lower educational attainment. These factors might have influenced their health trajectories and led to different associations with epigenetic aging. Despite these limitations, it’s essential to emphasize that the 2016 HRS epigenetic aging data offer valuable insights into potential associations, considering the varying life experiences and historical contexts across cohorts that could contribute to variations in observed effects. This snapshot of epigenetic aging in 2016 provides a natural history perspective on the experiences of children who either did or did not experience parental divorce and lived up to 2016 in a population-representative study. It is expected that as the population ages, the composition of birth cohorts changes, and contextual and social circumstances evolve with each cohort, findings may also evolve accordingly. Factors such as education and race/ ethnicity are known to differentially affect health trajectories and mortality. However, we include these as covariates and proposed mediators to account for their effects on the rate of epigenetic aging, which is our primary outcome. We believe that utilizing multiple years of epigenetic data in the future would offer a more comprehensive understanding of the interplay between age and cohort effects.

## Conclusion

Our study contributes to research on the relationship between parental divorce and health by showing differential effects of parental divorce and its pathways for different birth cohorts. It also contributes to research by documenting how early life events can be related to accelerated epigenetic aging, an underlying mechanism of aging, and its impact on health outcomes across the lifespan. The variation of these effects by cohort suggests that social and environmental contexts can shape these influences over time.

## Figures and Tables

**Table 1:** Descriptive Statistics (N = 1,545)

	Earlier Cohort born from 1916–1935 (N=470)	Later Cohort born from 1951–1965 (N = 1,075)
	Mean or %	Mean or %
Epigenetic Age	0.81 (SD = 0.16)	0.75 (SD = 0.16)***
Parental Divorce	9.81%	15.70%**
Chronic Depressive Symptoms	0.98 (SD = 0.97)	1.41 (SD = 1.75)***
Education: Years of Schooling (years)	12.72 (SD = 2.56)	13.85 (SD = 2.77)***
Cigarette Pack Years (pack years)	13.50 (SD = 19.01)	9.29 (SD = 17.89)***
Highest BMI (kg/m^2^)	29.35 (SD = 5.13)	31.36 (SD = 8.07)***
Divorce Experience	13.73%	20.98%**
Age (years)	85.82 (SD = 3.91)	60.23 (SD = 2.84)***
Female	62.63%	54.48%**
Race/Ethnicity		
White	88.90%	82.60%**
Black	6.99%	10.17%
Hispanic	4.10%	7.23%*

*** p < .001;

** p < .01;

* p < .05

**Table 2:** Regression Results of DunedinPACE on Parental Divorce and Mediating Characteristics

	Earlier Cohort				Later Cohort			
	M1		M2		M3		M4	
	b	95% CI	b	95% CI	b	95% CI	b	95% CI
Parental Divorce	0.637	−4.086, 5.360	0.211	−4.414, 4.835	2.876*	0.441, 5.311	0.923	−1.301, 3.147
Mediators								
Chronic Depressive Symptoms			1.069	−0.322, 2.461			0.114	−0.395, 0.623
Education			−0.347	−0.879, 0.184			−0.934***	−1.264, −0.604
Pack Years			0.093**	0.027, 0.159			0.158***	0.110,0.207
Highest BMI			0.350**	0.100, 0.599			0.691***	0.584, 0.799
Divorce Experience			2.613	−1.514, 6.740			0.730	−1.224, 2.683
Controls								
Age	0.008	−0.310, 0.326	0.064	−0.257, 0.385	−0.082	−0.411, 0.247	−0.021	−0.315, 0.273
Female	−1.420	−4.302, 1.462	−1.206	−4.176, 1.765	−1.749	−3.509, 0.010	−1.756*	−3.347, −0.165
Race/ethnicity (Non-Hispanic White as ref)								
Non-Hispanic Black	10.100***	4.620, 15.580	0.189**	2.621, 13.757	16.902***	13.968, 19.835	14.175***	11.527, 16.824
Hispanic	13 772***	6.666, 20.877	9.971*	2.428, 17.514	8.282***	4.899, 11.664	4.122*	0.918, 7.325
Constant	79.463***	52.237, 106.690	66.310***	34.610, 98.010	78.268***	58.313, 98.222	64.930***	46.356, 83.503
AdjR^2^	0.05		0.09		0.13		0.31	
Root Mean Squared Error (RMSE)	15.28		14.93		14.56		12.93	
Mean Absolute Error (MAE)	11.92		11.43		11.26		10.12	

Age, gender and race/ethnicity are controlled

*** p < .001;

** p < .01;

* p < .05

**Table 3:** Total Direct and Indirect Effect of Parental Divorce on DunedinPACE through Chronic Depressive Symptoms, Education, Pack years, BMI and Own Divorce for Earlier and Later Cohorts

	Earlier Cohort			Later Cohort		
	Standardized Coefficient	Bootstrapped SE	95% CI	Standardized Coefficient	Bootstrapped SE	95%CI
Indirect effect	0.024	0.017	−0.006, 0.060	0.060	0.016	0.029, 0.090
Chronic Depressive Symptoms	0.002	0.006	−0.007, 0.018	0.013	0.005	0.005, 0.023
Education	0.010	0.007	0.002, 0.027	0.014	0.005	0.004, 0.025
Pack Years	0.002	0.005	−0.007, 0.015	0.019	0.006	0.008, 0.031
Highest BMI	0.006	0.009	−0.010, 0.027	0.006	0.010	−0.014, 0.027
Divorce Experience	0.003	0.006	−0.010, 0.016	0.008	0.005	−0.000, 0.017
Direct effect	0.002	0.038	−0.069, 0.076	0.047	0.030	−0.012, 0.104
Total effect	0.026	0.042	−0.055, 0.109	0.106	0.031	0.046, 0.168
Proportion of total effect mediated	0.93			0.56		

Standard errors with 5,000 replications
